# Supplementary material for: Integrating genomic structural equation modeling and experimental validation to unravel the genetic basis of male genital lichen sclerosus
Source: Front Immunol. 2026 Jul 16;17:1857945. doi: 10.3389/fimmu.2026.1857945 (PMC13435250; doi:10.3389/fimmu.2026.1857945)
Supplement: Supplementary file 6 [file SupplementaryFile5.zip › result/fuma_PSYCH_factor_sCCA1_focus.chr7.chr7.149840658.150711505.pop1.pdf]

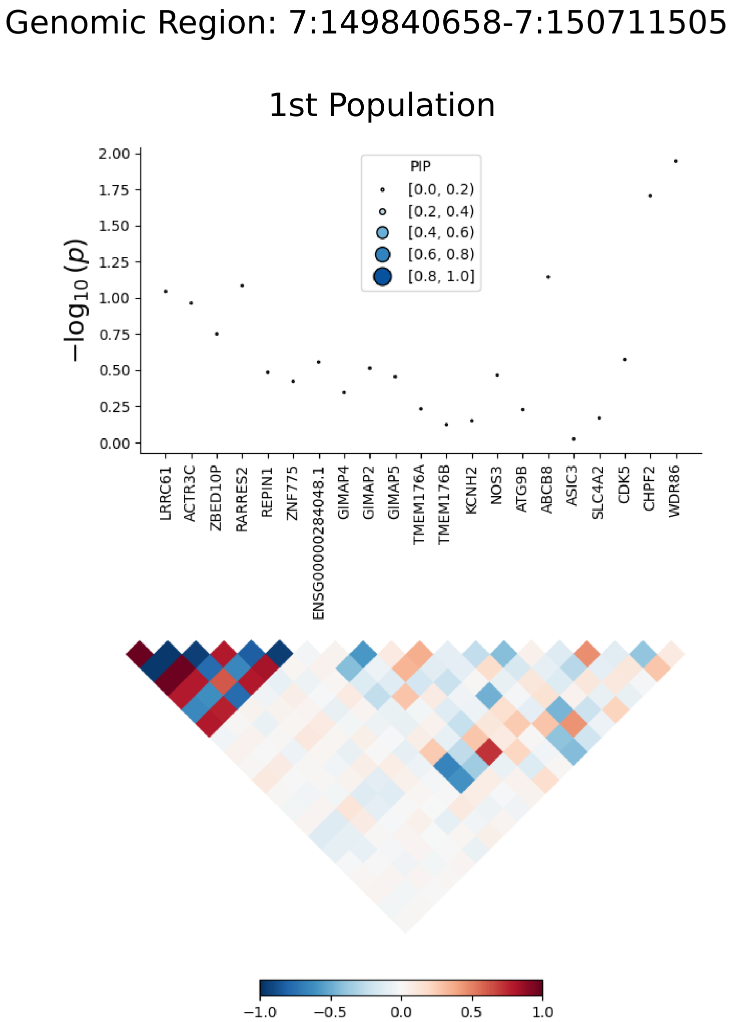
Genomic Region: 7:149840658-7:150711505
1st Population
PIP
[0.0, 0.2)
[0.2, 0.4)
[0.4, 0.6)
[0.6, 0.8)
[0.8, 1.0]
−log10(p)
2.00
1.75
1.50
1.25
1.00
0.75
0.50
0.25
0.00
LRRC61
ACTR3C
ZBED10P
RARRES2
REPIN1
ZNF775
ENSG00000284048.1
GIMAP4
GIMAP2
GIMAP5
TMEM176A
TMEM176B
KCNH2
NOS3
ATG9B
ABCB8
ASIC3
SLC4A2
CDK5
CHPF2
WDR86
−1.0
−0.5
0.0
0.5
1.0
